# Coronary calcium score in patients with post-surgical hypoparathyroidism

**DOI:** 10.20945/2359-4292-2023-0053

**Published:** 2024-04-09

**Authors:** Jhenifer Franco de Souza Sartori, Maria Luiza dos Santos, Aline Stollmeier, Rodrigo Julio Cerci, Carolina Aguiar Moreira, Victoria Zeghbi Cochenski Borba

**Affiliations:** 1 Universidade Federal do Paraná Curitiba PR Brasil Universidade Federal do Paraná, Curitiba, PR, Brasil; 2 Universidade Federal do Paraná Hospital de Clínicas Curitiba PR Brasil Hospital de Clínicas, Universidade Federal do Paraná, Curitiba, PR, Brasil; 3 Quanta Diagnóstico por Imagem Curitiba PR Brasil Quanta Diagnóstico por Imagem, Curitiba, PR, Brasil; 4 Universidade Federal do Paraná Divisão de Endocrinologia Departamento de Medicina Curitiba PR Brasil Departamento de Medicina, Divisão de Endocrinologia, Universidade Federal do Paraná, Curitiba, PR, Brasil

**Keywords:** Hypoparathyroidism, calcium supplementation, cardiovascular risk, coronary calcium score

## Abstract

**Objective:**

This study aimed to evaluate the cardiovascular risk of patients with post-surgical hypoparathyroidism through coronary calcium score (CACS) evaluation and cardiovascular risk calculators.

**Subjects and methods:**

Patients with post-surgical hypoparathyroidism (HG = 29) were compared to a control group (CG = 29), matched by sex and age. Demographic and clinical data were captured by a questionnaire or patient files. Both groups performed a thoracic-computed tomography to evaluate the CACS and the cardiovascular risk was calculated by two risk calculators.

**Results:**

In the HG, the supplementation of calcium varied between 500 to 2,000 mg/day and the mean calcitriol was 0.5 ± 0.29 mcg/day. The mean serum calcium and phosphorus were 8.32 ± 0.68 and 4.92 ± 0.87 mg/dL, respectively, and in the range recommended for hypoparathyroidism. The Brazilian Society of Cardiology's risk calculator showed a difference among groups, with no patient in the HG with low risk, but the CACS was similar. A positive CACS in the HG was associated with obesity and high BMI but not with calcium and/or vitamin D supplementation.

**Conclusion:**

In conclusion, patients with hypoparathyroidism did not show increased CACS, and it was not related to supplementation.

## INTRODUCTION

Hypoparathyroidism is a rare condition with a prevalence ranging from 6.4-37/100,000 population in different countries, with an incidence from 0.8-2.6/100,000/year (1,2). Low serum calcium levels, high serum levels of phosphorus, and deficient production of parathyroid hormone (PTH) characterize it (1,2). Post-surgical, after a total thyroidectomy or other anterior neck surgery, is the most common etiology, accounting for about 75% of the cases, followed by autoimmune disease (2,3).

Clinical manifestations of hypoparathyroidism are due to acute or chronic hypocalcemia and they include peripheral and central nervous symptoms, such as paresthesia, tetany, seizures, and intracerebral calcifications, mainly in the basal ganglia. Bone remodeling is reduced in hypoparathyroidism, with increased bone mineral density (4,5). Other long-term complications include renal dysfunction, nephrolithiasis, and cataracts (4,5).

The absence of PTH can reduce calcium coming from bone resorption and decrease 1,25-dihydroxyvitamin D production. Furthermore, a lack of PTH reduces renal excretion of phosphate, causing hyperphosphatemia, and consequently an increased calcium-phosphorus product, which could cause vascular calcification and increase the cardiovascular risk (3,6,7).

Although effective, PTH replacement is still not a viable option for hypoparathyroidism therapy (8); therefore, replacing calcium and the active form of vitamin D (1,25-dihydroxyvitaminD) are necessary. In this sense, the clinical treatment of hypoparathyroidism aims to correct the hypocalcemia and its symptoms, maintaining the serum levels of calcium in the lower limit of normal and phosphate in the upper normal, along with avoiding associated complications (9).

A significant number of patients need high doses of these drugs to reach the therapeutic goal; others cannot control their calcium levels or even cannot consistently stand high-dose therapy, causing variability of calcium levels during treatment. Calcium variability occurs during periods of excessive medication and could lead to hypercalcemia, increased risk of kidney disease, hypercalciuria, nephrolithiasis and nephrocalcinosis, and cardiovascular disease, despite periodic monitoring (4,9).

A high dose of calcium supplementation has been associated with increased cardiovascular risk in patients with osteoporosis (10,11). Extrapolating this data to patients with hypoparathyroidism who supplement with high doses of calcium, it becomes important to investigate these patients’ cardiovascular risk. In addition, a parallel between the levels of calcium and phosphorus in patients with chronic kidney disease (CKD) and their high cardiovascular risk, suggest that patients with hypoparathyroidism similarly could present an increased cardiovascular risk.

The coronary artery calcium score (CACS) is an important tool for analyzing subclinical atherosclerosis and thus, of cardiovascular risk because it identifies calcified plaques in the topography of coronary arteries. Furthermore, the CACS predicts, with diagnostic and prognostic value, risk stratification and cardiovascular disease, mainly in asymptomatic individuals (12).

Thus, this study aimed to evaluate the cardiovascular risk through the CACS and the cardiovascular risk calculators in patients with post-surgical hypoparathyroidism without known cardiovascular disease.

## SUBJECTS AND METHODS

This was a transversal controlled study of patients with hypoparathyroidism in treatment at the *Serviço de Endocrinologia e Metabologia do Hospital de Clínicas da Universidade Federal do Paraná* (SEMPR). The Human Research Ethics Committee of the hospital approved the project under the number 4.909.923; Certificate of Ethical Appreciation 30077720.4.0000.0096.

### Sample selection

An active search for patients with hypoparathyroidism was conducted in the SEMPR database. After selecting patients with post-surgical hypoparathyroidism, they were recruited during their routine visits to the clinic. Patients with post-surgical hypoparathyroidism (hypoparathyroidism group: HG) with at least 5 years of diagnosis were included. Those with established cardiovascular disease (coronary, cerebrovascular, or peripheral arterial disease); with comorbidities associated with high cardiovascular risk, such as uncontrolled arterial hypertension (blood pressure ≥ 140/90 mmHg) (13) and chronic kidney disease with a glomerular filtration rate lower than 45 mL/min/1.73 m³; systemic rheumatic disease; advanced stage cancer or in chemotherapy and/or radiotherapy in the last five years; chronic use of corticosteroids; pregnant women; and individuals younger than 18 years old were excluded. The selected patients who agreed to participate signed an informed consent form, answered a standardized questionnaire, and were referred to undergo a thoracic computer tomography exam on a previously scheduled day in a maximum period of 30 days.

The control group (CG) consisted of individuals followed in the same service who underwent total thyroidectomy and who did not have post-surgical hypoparathyroidism, matched by age and sex. The CG followed the same exclusion criteria and performed the same evaluations as the HG did.

### Study procedures

The standardized questionnaire included information on gender, age, ethnicity, current and previous smoking, and alcohol consumption. Participants’ current and past medical history, including comorbidities (presence of dyspnea and chest pain when exercising, presence of daily cough, orthopnea, paroxysmal nocturnal dyspnea, history of acute myocardial infarction, history of stroke, arrhythmia, heart surgery or angioplasty, osteoporosis, nephrolithiasis, chronic kidney disease or history of other kidney diseases, diabetes, hypertension, dyslipidemia); continuous medications in use (vitamin D, calcitriol and calcium replacement, antihypertensive drugs, antiresorptive, diuretics, statins, antiplatelet agents, anticoagulants, corticosteroids, and antidepressants); family history of acute myocardial infarction, stroke, and sudden death were also investigated. Information not captured with the questionnaire was obtained from medical records, and the patients clarified discordant information from medical records.

The laboratory tests closest to the evaluation (maximum of 6 months) were captured from patients’ charts and included serum calcium (normal value [NV]: 8-10.5 mg/dL], serum inorganic phosphate (NV: 3-4.5 mg/dL), 25-hydroxyvitamin D (25OHD) (NV: 30-60 ng/mL), PTH (NV: 10-65 pg/mL), urinary calcium (NV: <4 mg/kg/24 hours), total cholesterol (NV: <200 mg/dL), HDL-cholesterol (NV: >60 mg/dL), LDL-cholesterol (NV: <100 mg/dL), triglycerides (NV: <150 mg/dL), fasting glucose (NV: <100 mg/dL), glycated hemoglobin (NV: <6.4%), and creatinine (NV: 0.6-1 .2 mg/dL).

### Coronary artery calcium score

The CACS was calculated using high-resolution computer tomography of the chest without contrast, with axial tomographic sections limited to the heart, coronary arteries, and proximal portion of the great vessels, with prospective electrocardiographic coupling in a 256-detector tomography scanner (iCT Elite, Philips Medical Systems, Eindhoven, Netherlands). Coronary calcification was quantified using a dedicated workstation. The calcium score was calculated using Agatston and cols.'s (14) suggested algorithm, which quantifies the calcification of the following arteries: anterior descending and circumflex, branches of the left coronary artery, and the right coronary artery. The calcium score was interpreted according to the patient's sex and age, placing it in a percentile, as McClelland and cols. (15) and the Brazilian Guideline on Dyslipidemia and Prevention of Atherosclerosis (16) recommendation. Calcification is defined as a signal intensity above 130 Hounsfield units (HU), calculated from the Agatson score, in which values between 0-10 indicate minimal calcification, 11-100 mild, 101-400 moderate, above 400 is considered severe calcification, and greater than 1,000 is considered very severe (14,17). A positive CACS is defined as an Agatson score above zero. These absolute values can be adjusted according to sex and age, represented by percentiles, with an aggravating risk factor being a positive CACS greater than the 75th percentile for age or sex (18).

### Cardiovascular risk assessment

The cardiovascular risk was calculated using two tools: the calculator for cardiovascular risk stratification of the Brazilian Society of Cardiology (SBC) (18,19) and the American College of Cardiology Atherosclerotic Cardiovascular Disease Plus calculator (ASCVD) (20) as based on Lloyd-Jones and cols.'s (21) study.

The ASCVD calculator estimates the 10-year risk into low (less than 5% risk), borderline (5% to 7.4%), intermediate (7.5% to 19.9%), and high (risk greater than 20%). It is limited to people between 40 and 79 years old, with systolic blood pressure between 90-200 mmHg, diastolic blood pressure between 60-130 mmHg, total cholesterol between 130-320 mg/dL, high-density lipoprotein (HDL) 20-100 mg/dL, and low-density lipoprotein (LDL) 30-300 mg/dL (21).

The SBC cardiovascular risk stratification calculator considers four stages, assesses the presence of atherosclerotic disease, diabetes, subclinical atherosclerosis, abdominal aortic aneurysm, chronic kidney disease, total cholesterol, LDL and HDL, sex, age, systolic blood pressure (treated or not), smoking, and statin use. The calculator stratifies patients into low, intermediate, high, and very high risk. It is limited to patients aged 30 years or more.

Cardiovascular risk was assessed for all patients and controls using all the required information and appreciation of the calculators’ limitations.

### Statistical analysis

In the inferential statistical analysis for the comparison between groups, the following non-parametric tests were used: Mann-Whitney and Fisher's exact tests for nominal data and chi-square for ordinal data. For parametric variables with normal distribution, the t-student test was used. Comparisons between variables and altered calcium scores were performed using the Mann-Whitney and Fisher's exact tests. Qualitative results were expressed as absolute frequency and percentage, and quantitative variables as mean and standard deviation (SD). P values < 0.05 are considered significant. All analyses were performed using the statistical software R (R Core Team, 2021) version 4.1.2. (22).

## RESULTS

All patients with post-surgical hypoparathyroidism (HG) followed up at SEMPR were initially selected. A total of 100 patients were identified, 71 were excluded and 29 patients were studied.

**Figure 1:**
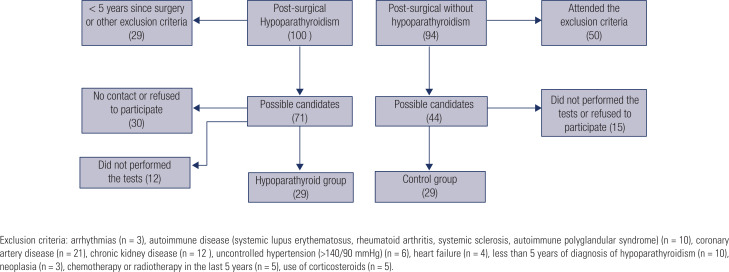
Selection of patients and controls.

A total of 94 patients who underwent cervical surgery and hypoparathyroidism were recruited as a control group (CG), 65 were excluded and 29 were included in the CG (Figure 1).

The final sample consisted of 58 individuals, 29 in each group. The mean age was 56.48 ± 14.35 years with the majority female and white (79.31%) (Table 1). Time since surgery was greater in the HG (11 years) compared to CG (5-10 years) (p < 0.001). Thyroid cancer was the most frequent indication for surgery in both groups, with greater proportion in the CG (p = 0.008).

**Table 1:** Demographic characteristics of the sample

Variables n (%)		HG (n = 29)	CG (n = 29)	p-value
Sex				1
	Female	25 (86.21%)	24 (82.76%)
	Male	4 (13.70%)	5 (17.24%)
Age (years), mean ± SD		57.90 ± 15.28	55.07 ± 13.35	0.200
Race, n (%)				0.004
	White	28 (96.55%)	18 (62.07%)	
	Black	1 (3.45%)	11 (37.89%)	
Smoking				
	Current	3 (10.34%)	2 (6.89%)	0.224
	Past	9 (31.03%)	5 (17.24%)	0.643
BMI (kg/m²), mean ± SD		28.69 ± 6.51	28.70 ± 3.94	0.470
Obesity		7 (26.9%)	8 (27.5%)	0.415
Menopause		14	15	0.107
	Hormone therapy	2	1	
Time since surgery				<0.001
	5-10 years	2 (8.69%)	18 (62.07%)	
	11-20 years	10 (43.48%)	9 (31.03%)	
	>20 years	11 (47.83%)	2 (6.9%)	
Reason for surgery				0.008
	Cancer	13 (56.52%)	23 (85.2%)	
	Graves disease	0	1 (3.7%)	
	Multinodular goiter	8 (34.78%)	1 (3.7%)	
	Functioning goiter	0	1 (3.7%)	
	Adenoma	2 (8.7%)	1 (3.7%)	
Family history				
	Myocardial infarction	3 (10.34%)	10 (34.48%)	0.050
	Sudden death	2 (6.89 %)	1 (3.34%)	1
	Stroke	6 (20.68%)	6 (20.68%)	1
Comorbidities				
	Hypertension	18 (62.06%)	19 (65.51%)	1
	Dyslipidemia	13 (44.82%)	15 (51.72%)	0.590
	Prediabetes/T2 DM	6 (20.68%)	11 (37.93%)	0.153
	Obesity	7 (24.10%)	8 (27.58%)	1
	Osteoporosis	5 (17.24%)	2 (6.89%)	0.420
	Nephrolithiasis	3 (10.34%)	2 (6.89%)	1
	Nephrocalcinosis	3 (10.34%)	0	0.230
	Arrhythmia	2 (6.89%)	1 (3.34%)	1
	Chronic kidney disease	2 (6.89%)	0	0.490
	Pulmonary disease	0	2 (6.89%)	0.490

MI: myocardial infarction; BMI: body mass index; T2DM: type 2 diabetes.

The family history of cardiovascular disease was also similar between groups, with a tendency to have a higher history of myocardial infarction in the CG (HG, 10.34% *vs.* CG, 34.48%, p = 0 .05), other comorbidities were comparable between them.

As expected, the laboratory profile of bone metabolism as serum levels of calcium, phosphorus, and PTH differed between HG and GC (p < 0.05). The other tests were similar (Table 2).

**Table 2:** Laboratory test and medication in use

Variables		HG (n = 29)	Controls (n = 29)	p-value
Laboratory (mean ± SD)				
Calcium (mg/dL)		8.32 ± 0.68	9.21 ± 0.50	<0.001
Phosphorus (mg/dL)		4.92 ± 0.87	3.53 ± 0.74	<0.001
25OHD (ng/mL)		39.36 ± 9.14	33.10 ± 14.10	0.010
PTH (pg/mL)		7.13 ± 3.71	55.05 ± 19.99	<0.001
Urinary calcium (mg/kg/d)		2.51 ± 1.00	3.99 ± 2.14	0.500
Total cholesterol (mg/dL)		191.31 ± 37.81	192.79 ± 39.31	0.880
LDL-cholesterol (mg/dL)		115.50 ± 32.19	120.97 ± 32.65	0.520
HDL-cholesterol (mg/dL)		49.21 ± 11.05	48.07 ± 9.34	0.670
Triglycerides (mg/dL)		123.62 ± 53.33	128.48 ± 68.31	0.810
Fasting glucose (mg/dL)		92.62 ± 8.12	104.59 ± 24.81	0.010
Glycated haemoglobin (%)		5.47 ± 0.42	5.96 ± 1.07	0.170
Creatinin (mg/dL)		0.89 ± 0.16	0.85 ± 0.17	0.350
Calcium-phosphate product		40.5 ± 5.80	33.36 ± 7.56	0.0001
**Medications**, n (%)				
Calcitriol (n %)		27 (93.1%)	-	
Calcitriol (mcg/day)		0.5 ± 0.29	-	
Calcium				<0.001
	0	3 (10.34%)	22 (75.86%)	
	≤500 mg	9 (31.03%)	5 (17.24%)	
	501-1,000 mg	8 (27.59%)	2 (6.9%)	
	1,001-2,000 mg	8 (27.59%)	0	
	>2,000 mg	1 (3.45%)	0	
Vitamin D		15 (51.72%)	12 (41.37%)	0.59
Antiresorptives		5 (17.24%)	2 (6.89%)	0.61
Antihypertensives		18 (62.06%)	19 (65.51%)	0.59
Diuretics		11 (37.93%)	9 (31.03%)	0.78
Statins		11 (37.93%)	12 (41.37%)	1

PTH: parathohormone; 25OHD: 25-hydroxivitamin D.

The HG took a median daily dosage of 750 mg (0-4,000 mg/day) of a calcium supplement and the majority (89.66%) took between 500 to 2,000 mg of calcium per day, which is higher than the median daily supplementation in the CG 0 (0-1,000 mg/day), where the majority (75.86%) did not supplement calcium (p < 0.001). In the HG, 93.1% took calcitriol, an average of 0.5 mcg/day (0 to 1.25 mcg/day) and none in the CG. The other medications in use did not differ between groups, as shown in Table 2.

Due to the calculators’ limitations, not all patients had the cardiovascular risk assessment performed. The ASCVD risk was performed in 21 patients of HG and 18 patients of CG, with a similar risk calculated between them. The SBC risk was calculated in 24 patients of HG and 21 of CG; there was a difference in risk distribution between HG and CG with no patients in the HG classified as low risk compared to 20% of the CG (p = 0.03). The HG had more patients at intermediate and high risk and no patients at low risk, while the high risk was similar between the groups.

Although the mean CACS and the percentage of positive CACS were higher in the HG, there was no difference between groups (p > 0.05) (Table 3).

**Table 3:** Cardiovascular risk score and coronary arterial calcification score

Variable		HG	CG	p-value
ASCVD (mean ± SD)				
Risk %		10.86 ± 9.45	10.19 ± 12.81	0.400
ASCVD, n (%)				0.970
	Low	6 (28.57%)	8 (36.36%)	
	Borderline	6 (28.57%)	5 (22.73%)	
	Intermediate	6 (28.57%)	6 (27.27%)	
	High	6 (28.57%)	3 (13.64%)	
SBC risk calculator, n (%)				
	Low	0	5 (20%)	0.030
	Intermediate	7 (30.43%)	3 (12%)	
	High	16 (69.57%)	17 (68%)	
	Very high	_	_	
CACS (mean ± SD)		32.55 ± 41.53	23.59 ± 36.92	0.390
Positive CACS, n (%)		7 (24.14%)	5 (17.24%)	0.580

ASCVD: atherosclerotic cardiovascular disease calculator; SBC: Brazilian Society of Cardiology calculator; CACS: coronary arterial calcification score.

An association of positive CACS with higher BMI and the diagnosis of obesity (p = 0.04 and p = 0.05, respectively) in the HG were observed. The univariate analysis considering the association of positive CACS and all other variables (serum calcium, 25OHD, PTH, 24 hours urinary calcium, calcium and vitamin D supplementation, use of antihypertensives, diuretics, and statins) did not show any difference in HG (p > 0.05).

The cardiovascular risk calculators ASCVD and SBC did not differentiate between HG patients with positive or negative CACS (p = 0.52 and p = 0.36, respectively) (Table 4).

**Table 4:** Association of the coronary arterial calcification score with cardiovascular risk calculators in the hypoparathyroidism group

Variable		Positive CACS (n = 7)	Negative CACS (n = 22)	p-value
ASCVD risk%, mean ± SD		11.27 ± 10.05	10.40 ± 9.25	0.430
ASCVD risk score, n (%)				0.520
	Low	3 (42.86%)	3 (21.43%)	
	Borderline	1 (14.28%)	4 (28.57%)	
	Intermediate	3 (42.86%)	4 (28.57%)	
	High	0	3 (21.43%)	
SBC risk score, n (%)				0.360
	Low	0	0	
	Intermediate	1 (14.28%)	6 (37.5%)	
	High	6 (85.72%)	10 (62.5%)	

CACS: Coronary artery calcium score; ASCVD: Atherosclerotic cardiovascular disease; SBC: Brazilian Society of Cardiology.

## DISCUSSION

This study showed that patients with hypoparathyroidism under conventional therapy had the same CACS as controls did, despite the higher supplementation of calcium and vitamin D. Laboratory tests were compatible with the diagnosis of hypoparathyroidism without an association between serum calcium and vitamin D levels and a high calcium score in patients with hypoparathyroidism.

Patients with hypoparathyroidism are recommended to keep calcium levels slightly below or at the lower limit of normal (8.0-8.5 mg/dL) and calcium-phosphorus product less than 55 to prevent ectopic calcification in the brain, kidney, and cardiovascular system (23). Thus, a possible explanation for low indices of altered calcium score in the HG could be due to an adequate mean serum calcium level of 8.32 ± 0.68, which is within the limits to prevent cardiovascular complications. According to Underbjerg and cols.'s study, extreme variations in serum calcium can affect the cardiovascular system; frequent episodes of hypercalcemia were associated with increased cardiovascular risk (6). This finding corroborates the orientation of keeping calcium levels within the lower normal limit.

Time after thyroidectomy was associated with higher cardiovascular risk (6), which we did not confirm in this study.

Evaluating a different population of 74,245 women using oral calcium supplementation (more than 1,000 mg) without a history of cardiovascular disease, cancer, or hypoparathyroidism in a 24-year prospective study, no increase in cardiovascular risk was observed, including coronary heart disease or stroke, agreeing with our study, which did not find significant association between oral calcium and/or vitamin D supplementation and altered calcium score (24).

Although rare, cardiac arrhythmias may be present in these patients, with QT interval prolongation on the electrocardiogram, in addition to a prominent U wave and T wave abnormalities (3). However, in this study, only two patients (6.89%) with hypoparathyroidism had a previous diagnosis of arrhythmia. The cardiac effects of this condition probably result from hypocalcemia through the reduction of cardiac contractility, but it remains a poorly understood mechanism (25). PTH exerts direct effects on the cardiovascular system through the PTH-1 receptor, which is expressed in endothelial and vascular smooth cells, cardiomyocytes, and the cardiac conduction system (26). As expected, we found low levels of PTH in the HG, and a slightly higher mean in the CG, without difference in presence of arrythmia. In contrast to this result, Underbjerg and cols. (26) compared 30 patients with hypoparathyroidism of non-surgical etiology with a group with pseudohypoparathyroidism, characterized by high levels of phosphate and PTH, and low levels of calcium, and found a higher pulse velocity in the hypoparathyroid group (p = 0.02) even after correcting for confounding factors, reinforcing the arterial stiffness of the first group, associated with a higher cardiovascular risk.

As in the present article, a case-controlled study evaluating 688 patients with hypoparathyroidism, showed there was no increase in cardiovascular risk in patients with post-surgical hypoparathyroidism (27). In contrast, others had demonstrated increased cardiovascular risk in patients with non-surgical hypoparathyroidism (28,29). Thus, it would be interesting to investigate the CACS in patients with hypoparathyroidism of other etiologies and longer disease progression to verify the correlation with cardiovascular risk. A possible hypothesis to justify this difference between the groups would be that the group with low PTH levels may have greater variation in calcium levels due to oral supplementation; therefore, transient hypercalcemia may increase the calcium–phosphorus product and promote vascular calcification (7).

In the literature, only one study investigated the CACS in patients with hypoparathyroidism, although it was performed in patients with idiopathic etiology. They found three (10%) altered scores, but no control patient had calcification on examination (p = 0.07), and the altered CACS associated only to lower calcium levels (30). Their results were similar to ours, except we did not find an association between laboratory data and positive CACS. The only significant association found in our study was in the HG, a positive CACS with high BMI and obesity, which were factors already associated with positive CACS in non-hypoparathyroid patients (31). A possible reason for the absence of difference between the CACS of patients and controls may be the presence of a non-significant higher number of patients with diabetes in the CG. However, this group had more patients classified with low cardiovascular risk per the SBC risk calculator.

Other studies described in patients with idiopathic hypoparathyroidism an increase in the intima thickness of the carotid artery (7) and carotid, aortic and renal arteries (30,32), thus reinforcing the difference among the etiologies of hypoparathyroidism. One possible explanation could be the shorter time for the diagnosis of post-surgical hypoparathyroidism compared to other etiologies that expose patients to a longer duration of hypocalcemia and hyperphosphatemia as well as the complications. Even though in this study we found a difference in the calcium product between groups, it was below the cut point of 55 in both, which could explain the lack of difference in the CACS score.

Limitations of this study include a lack of data on dietary calcium intake, physical activity, and time since menopause, in addition to the limited information obtained from the medical records. The cardiovascular risk calculators had limitations, adding to possible confounding factors that may have restricted the results. The study's strengths are the small sample size concerning the general population, but significant for hypoparathyroidism, a rare disease; the assessment of the CACS, an important tool to estimate the cardiovascular risk of all patients; and the inclusion of a control group that performed the same surgery as the HG.

In conclusion, this study did not find a difference in the CACS of patients with post-surgical hypoparathyroidism compared to controls or the two cardiovascular risk calculators. Using the SBC calculator, we showed there were more low-risk patients in the CG compared to the HG, although there was no between-group difference on the CACS or on the ASCVD calculator. Thus, further studies may be conducted in the future, with large-scale prospective follow-up to verify broadly the possible cardiovascular outcomes in patients with hypoparathyroidism, involving all etiologies, based on the CACS.
